# Efficacy of Submucosal Injection of Chymotrypsin, Oral Serratiopeptidase or Oral Dexamethasone in Reducing Postoperative Complications Following Impacted Lower Third Molar Surgery: A Prospective, Randomized, Double-Blind, Controlled Clinical Trial

**DOI:** 10.3389/froh.2020.575176

**Published:** 2020-12-08

**Authors:** Essam Ahmed Al-Moraissi, Elham Aziz Al-Zendani, Abeer Mohammed Al-Selwi

**Affiliations:** ^1^Department of Oral and Maxillofacial Surgery, Thamar University, Dhamar, Yemen; ^2^University of Science and Technology, Sanaa, Yemen

**Keywords:** chymotrypsin, oral serratiopeptidase, postoperative complications, dexamethasone, lower third molars surgery

## Abstract

**Aim** The study aimed to compare between chymotrypsin, oral serratiopeptidase, and oral dexamethasone following impacted mandibular third molars surgery in respect of postoperative complications.

**Materials and method:** A randomized, double-blind clinical trial was conducted on 60 patients who were candidates for impacted mandibular third molars surgery and randomly allocated into the following 3 groups: submucosal chymotrypsin (5 mg), oral serratiopeptidase (10 mg), and oral dexamethasone (8 mg) (each group = 20). The outcome variables were postoperative pain (via visual analog scale), facial swelling (via tape method) and maximal mouth opening immediately after 2nd, 3rd, and 5th postoperative days.

**Results:** A total of 60 patients underwent randomization and allocation concealment and were included in the current study. All of the subjects tolerated the medicines with no untoward side or adverse effects. There was no statistically significant difference between the three groups in respect of postoperative pain intensity, facial swelling and maximal mouth opening at the immediate first hour, 2nd, 3rd, and 5th postoperative days (*P* < 0.05).

**Conclusion:** The present randomized clinical trial concluded that preemptive sub-mucosal injection of chymotrypsin yields a comparable effectiveness in decreasing postoperative sequelae following impacted mandibular third molars surgery when compared to oral serratiopeptidase or dexamethasone. This is the first Randomized Clinical Trail that assessed efficacy and safety of sub-mucosal injection of chymotrypsin after impacted mandibular third molars surgery. This trial is registered at clinicaltrials.in.th, number (TCTR20200828006).

## Introduction

Surgical extraction of the impacted mandibular third molars is the most common procedure in oral surgery clinics [1]. Most of the common postoperative complications following lower third molars surgery are a pain, trismus, and facial swelling. Adequate surgical methods such as selecting an appropriate flap design, minimal bone removal and less trauma to adjacent soft tissues with proper wound closure techniques could decrease the incidence of postoperative sequelae, but not eliminate it [2]. Therefore, several pharmacologic medications have been reported to be used as maneuvers to control the postoperative sequelae after lower third molar surgery includes: Non-steroidal anti-inflammatory drugs (NSAIDs) [3, 4], and corticosteroids [5, 6], along with routine antibiotics [7, 8].

Because conventional anti-inflammatory medications (steroid and NSAIDs) are associated with several adverse effects, natural anti-inflammatory proteolytic enzymes, such as trypsin, chymotrypsin, papain, serratiopeptidase, and bromelain have been used following lower third molars surgery [9–11].

Serratiopeptidase is a proteolytic enzyme formed by Enterobacterium Serratia, which has substantial anti-inflammatory and pain relieving action [12, 13]. Similarly, chymotrypsin and trypsin, bromelain (pineapple enzyme), and papain are other proteolytic enzymes have been taken to decrease inflammation, reduce edema and accelerate healing [14–17].

Several randomized clinical trials have reported the beneficial effects of proteolytic enzymes in reducing postoperative complications following third molar surgery [9–11, 15, 18, 19].

Several studies have investigated submucosal, intramuscular injection and oral administration of dexamethasone after lower third molars surgery, showing a significant role in the reduction of postoperative pain, trismus, and edema [6, 20–25]. However, the comparison between sub-mucosal injection of chymotrypsin, oral serratiopeptidase and dexamethasone after lower third molars surgery have not yet been investigated. Authors hypothesized that sub-mucosal injection of chymotrypsin, oral serratiopeptidase would produce a superior pain, fascial swelling and mouth opening reduction after lower third molars surgery when compared to oral dexamethasone. So, the study aimed to compare between chymotrypsin, oral serratiopeptidase and oral dexamethasone following lower third molars surgery in respect of postoperative complications.

## Materials and Methods

### Study Design

A randomized, double-blind controlled, clinical study was performed conforming to the consolidated standards of reporting trials [26] statement and the Declaration of Helsinki. Ethical approval was given before the commencement of study from the Scientific Research Committee of at the Faculty of Dentistry, University of Technology and Sciences, Sanaa, Yemen. All patients were informed about everything concerning the study steps such as randomization, blinding for assessor and surgical extraction procedures. Patients had the right to withdraw from the trial at any time. This trial is registered at clinicaltrials.in.th, number (TCTR20200828006).

### Sample Size Calculation

Sample size calculation was computed for the outcome of postoperative pain intensity after lower third molar surgery. In a previous study, the response within the control subject (dexamethasone) per 2 experimental subjects. In a previous study [27] the response within each subject group was normally distributed with a standard deviation of 1.68916. If the true difference in the experimental and control means is 2.521, so we needed to study 40 experimental subjects (2 groups, each group composed of 20 patients) and 20 control subjects to be able to reject the null hypothesis that the population means of the chymotrypsin, and serratiopeptidase and dexamethasone groups were equal with probability (power) 0.8. The type I error probability associated with this test of this null hypothesis is 0.05.

### Randomization

A random allocation sequence was generated using the computerized method (https://www.randomizer.org/). Then, allocation concealment was done via an opaque sealed envelope to prevent selection bias in the recruitment stage. Both generations of random sequence and allocation concealment were achieved prior to the beginning of the study by the first author (E.A). All medications (serratiopeptidase, dexamethasone, and corticosteroids tablets) were given to patients with special sterilized bottles without identification of nature and name of medicine. Additionally, postoperative assessment of outcomes was done by the blinded assessor (AA). Thus, this study was double blind for patients and assessor.

### Inclusion Criteria

1. Adult healthy patients who were American Society of Anesthesiologists (ASA) group I and required surgical extraction of unilateral or bilateral complete impacted mandibular third molars.
2. All subjects had to presented with the same surgical difficulty concerning similar bone impaction and had the same classification in relation to the occlusal surface of the neighboring second molar (Class B: the impacted teeth are partly buried in the bone, or the occlusal plane of the impacted tooth is between the occlusal plane and the neighboring tooth's cervical line).

### Exclusion Criteria

1. Patient administered other drugs such as NSAIDS and steroids.
2. Patient has allergy to the drugs used in this study.
3. Pregnant patient or a patient with lactation.
4. Immunocompromised patients with diabetic or hypertension (from patient's history).
5. Patients with irradiated maxillofacial region.
6. Intellectually disabled patients and patients unable to come for follow up visits.
7. Patients with acute and subacute pericoronitis

Subjects fulfilling the inclusion criteria were randomly divided into the following groups: [1] Chymotrypsin group: consisting of 20 patients who received a pre-operative sub-mucosal injection of 5 mg chymotrypsin (Alfa Chymotrypsin) at the pterygo-mandibular space following the inferior alveolar nerve block; [2] Oral serratiopeptidase group: consisting of 20 patients who received 10 mg oral serratiopeptidase (Cipzen Forte) at immediate post-operative time and twice a day for 5 days post-operative; and [3] Dexamethasone group: consisting of 20 patients who received 8 mg oral Dexamethasone at immediate post-operative time and twice a day for 5 days post-operative.

### Pre-operative Assessments

#### Maximal Mouth Opening

Distance between the incisal edge of the upper central incisors and the incisal edge of the lower central incisors in the maximum opening by a boley gauge caliper was measured preoperatively [28].

#### Facial Swelling

**Figure 1:**
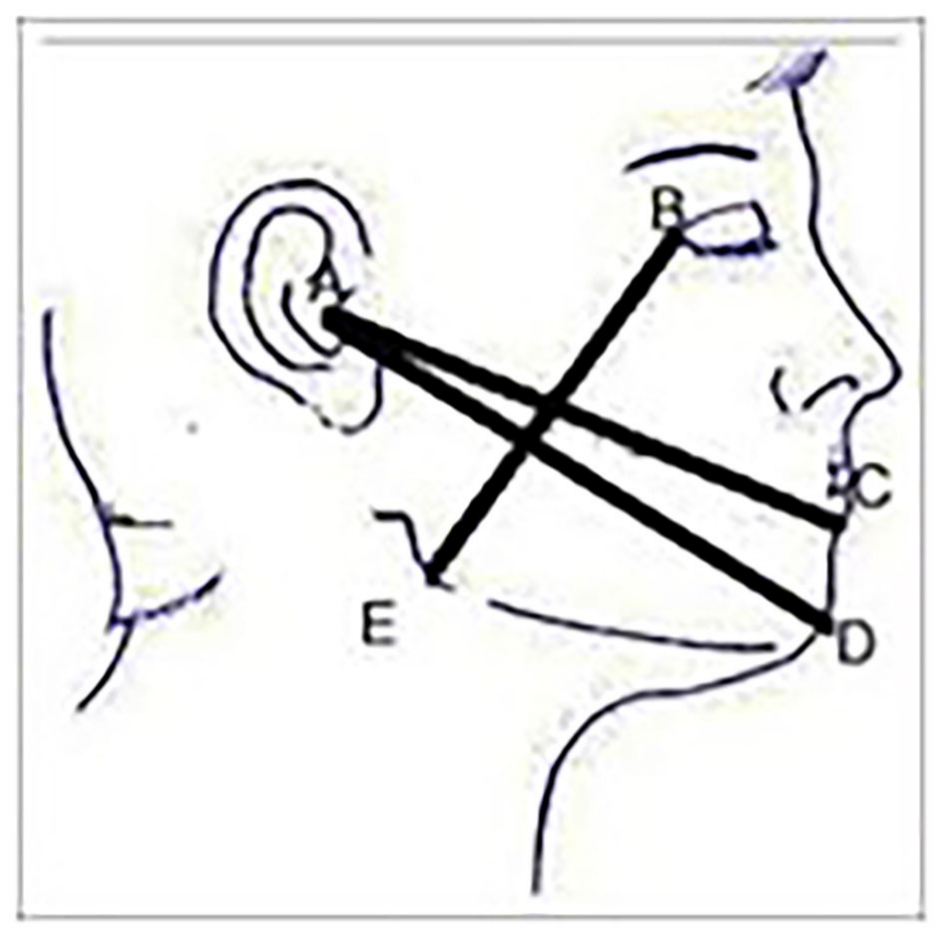
Measurement of facial swelling using tape method.

Facial swelling on the operated side was measured prior to surgical extraction using three lines namely: tragus to soft tissue pognion, tragus to corner of the mouth and gonion to lateral canthus, using a tape measure [28] (Figure 1).

#### Pain Intensity

Pain intensity was evaluated using the Visual Analog Scale (VAS) 10 mm long, that ranged from 0 = no pain to 10 = the worse possible pain [29].

### Surgical Technique

Panoramic and pri-apical radiographs were taken to assess the third molar positions and to check the presence of any pathological lesion. Surgical extraction was performed by the same surgeon using a standardized technique[9] with the following steps: [1] Standard anesthesia of inferior alveolar nerve block and the long buccal nerve block using a solution of 2%lognocaine hydrochloride and epinephrine 1:100,000; [2] A triangular full-thickness mucoperiosteal flap with releasing incision on the disto-buccal aspect of the second molar; [3] Bone removal around the tooth with straight hand-piece under continuous irrigation with normal saline; [4] Tooth sectioning when necessary and gently elevated; [5] Sockets inspected and irrigated copiously with normal saline; [6] The flap suture back with interrupted 3-0 silk sutures; 7) Small gauze packs applied to the site and usual post-operative instructions were given to the patients.

### Postoperative Assessment

Measuring the maximal interincisal opening, facial swelling and pain intensity were made immediately after the procedure, and on 1st, 2nd, 3rd and 5th postoperative days.

### Statistical Analysis

Descriptive statistics and frequency distributions were performed on the preoperative measurements. Independent sample Kruskal-Wallis equality-of-populations rank test or one-way ANOVA test were used to compare groups continuously according to the distribution of data. Chi-square test was used to compare categorical variables. Repeated measures analysis of variance was used to evaluate the change over time and differences between groups for the outcomes of interest. Analysis of mean response profile was done using the generalized estimated equation to assess the effect of group, time, and group time interaction. All analyses were conducted using STATAIC/15.1. A *p* < 0.05 indicated statistical significance.

## Results

A total of 60 patients underwent randomization and allocation concealment and were included in the current study. There were no dropouts. All of the subjects accepted the medicines appropriately with no untoward side or adverse effects. Surgical extraction sites were healed uneventfully. The mean age of all patients was 29.13 ± 8 (range 19–39 years). Twenty five patients (41.6%) were males and 35 patients (58.3%) were females. Right side impaction was more frequent (53.3%) than left side impaction (46.6%). There were 36.6% mesioungular, 31.6% vertical and 31.6% horizontal (Tables 1 and 2).

**Table 1:** The population characteristics.

**Variable**	**Mean (SD)**	**Range**
Age	29.13 (8)	(19-39)

**Table 2:** Characteristics of the patients.

**Variable**		**Number**	**Percent**
Gender (Male/Female)		25/35	41.6/58.3
Location of impaction (R/L)		32/28	53.3/46.6
	Mesioangular	22	36.6%
Type of impaction	Vertical	19	31.6%
	Horizontal	19	31.6%

### Baseline Characteristics Among the Three Groups

Demographic and baseline characteristics of the study groups showed in Table 3.

**Table 3:** Population characteristics of the three groups.

**Variable**	**Group A**	**Group B**	**Group C**	***P*-value**
Age Median (IQR)	25.5 (21–29.5)	28.5 (24–37)	29 (24–35)	0.18
Gender M/F	45/55%	40/60%	40/60%	0.710
Preoperative pain Median (IQR)	1 (0–3)	0 (0–2)	1 (0–2)	0.33
Preoperative mouth opening Mean (SD)	4.06 (0.68)	4.16 (0.78)	3.81 (0.66)	0.41^***^
Preoperative facial swelling (tragus to pogonion) Median (IQR)	12.405 (1.5)	12.215 (1.568)	11.895 (1.58)	0.5791
Preoperative facial swelling (tragus to mouth) Median (IQR)	11.51 (1.54)	10.77 (1.45)	10.525 (1.70)	0.784
Preoperative facial swelling (gonion to canthus) Median (IQR)	11.315 (2.36)	9.44 (1.42)	9.63 (1.83)	0.096

*** *P-value=1.000 between groups A and B, 0.040 between groups A and C, 0.002 between groups B and C*.

*A: Submucosal chymotrypsin group, B: Oral serratiopeptidase group, C: Dexamethasone group*.

### Outcomes Variables

#### Postoperative Pain Intensity

**Figure 2:**
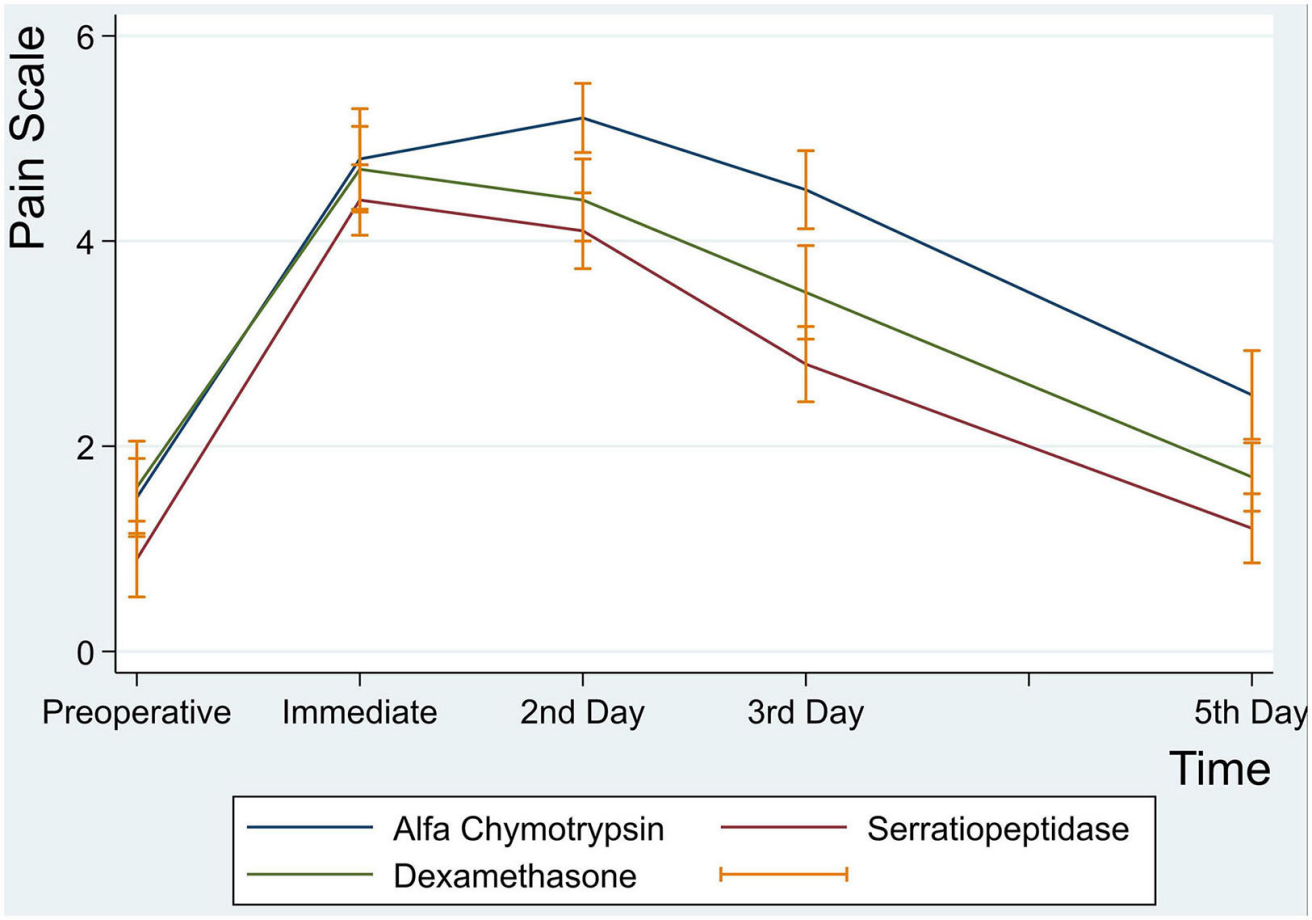
Mean pain intensity scores at preoperative, immediate, 2nd, 3rd and 5th postoperative days.

There were no statistically significant differences in postoperative pain intensity among the three groups at immediate first hour, 2nd, 3rd, and 5th postoperative days (*p* > 0.05) (Table 4 and Figure 2).

**Table 4:** Comparison of pain score (VAS) experienced by study groups.

	**Coefficient (95% Conf. Interval)**	***P*-value**
**Group**	−0.10 (−0.48 – 0.29)	0.62
A vs. B	−0.68 (−1.44 – 0.08)	0.08
A vs. C	−0.21 (−0.58 – 0.16)	0.27
B vs. C	0.47 (−0.26 – 1.21)	0.21
Time	0.07 (−0.20 – 0.34)	0.61
Group and time interaction	−0.09 (−0.21 – 0.04)	0.16

*A: Submucosal chymotrypsin group, B: Oral serratiopeptidase group, C: Dexamethasone group*.

#### Postoperative Maximal Mouth Opening

**Figure 3:**
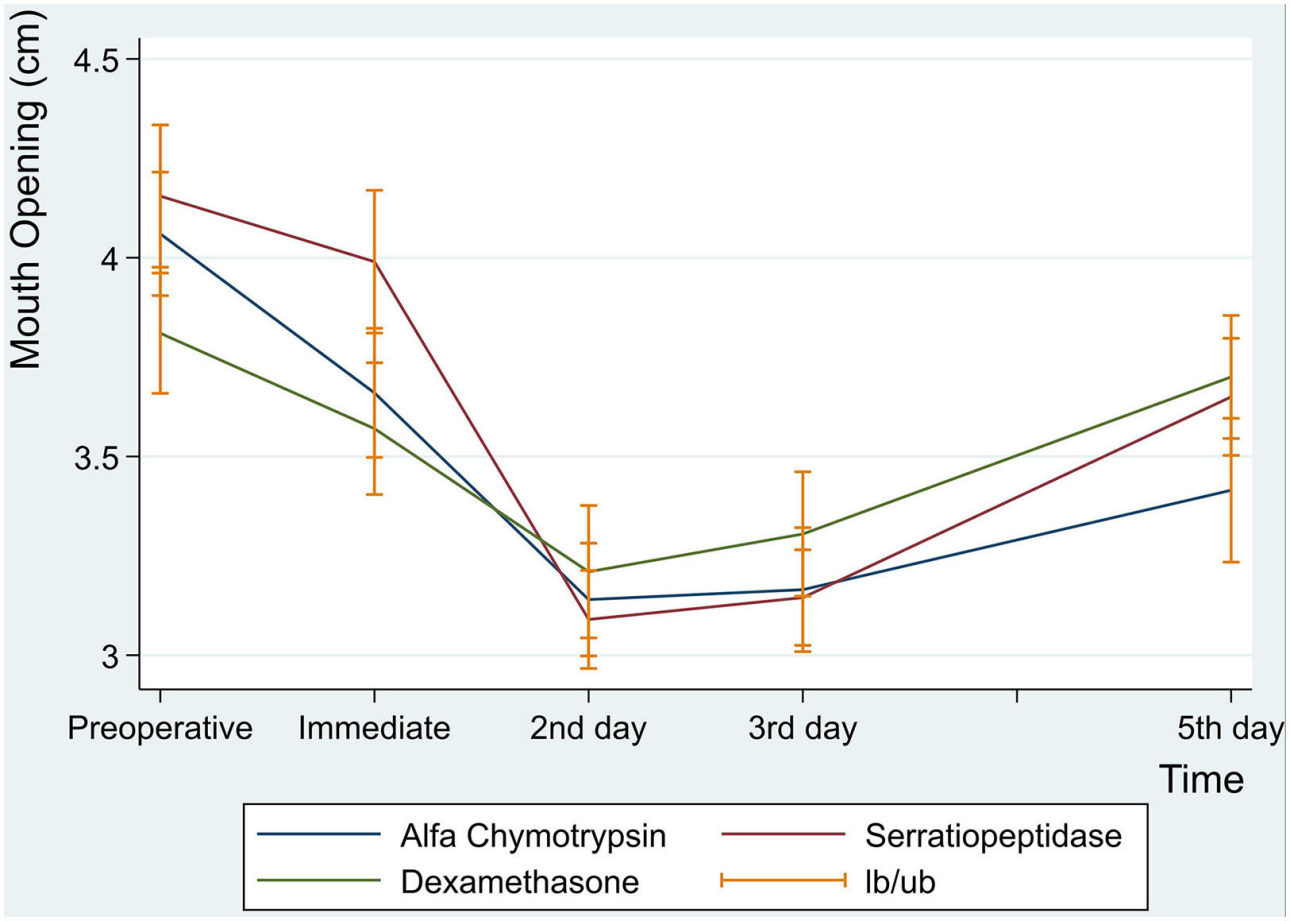
Maximal mouth opening at preoperative, immediate, 2nd, 3rd and 5th postoperative days.

Postoperative maximal mouth opening showed a sharp decrease in the first day following surgery in the three groups, then increased gradually from the second to the fifth postoperative days. There was no significant difference in postoperative maximal mouth opening among the three groups. However, an interaction between groups and time was a statistically significant (*p* = 0.001) (Table 5 and Figure 3).

**Table 5:** Comparison of mouth opening experienced by study groups.

	**Coefficient (95% Conf. Interval)**	***P*-value**
Group	−0.09 (−0.28 – 0.09)	0.315
A vs. B	−0.00 (−0.35 – 0.34)	0.99
A vs. C	−0.14 (−0.32 – 0.05)	0.16
B vs. C	−0.09 (−0.45 – 0.27)	0.62
Time	−0.14 (−0.20 – −0.07)	0.000
Group and time interaction	0.05 (0.02 – 0.08)	0.001

*A: Submucosal chymotrypsin group, B: Oral serratiopeptidase group, C: Dexamethasone group*.

### Postoperative Facial Swelling

#### Facial Swelling (Tragus to Pogonion)

**Figure 4:**
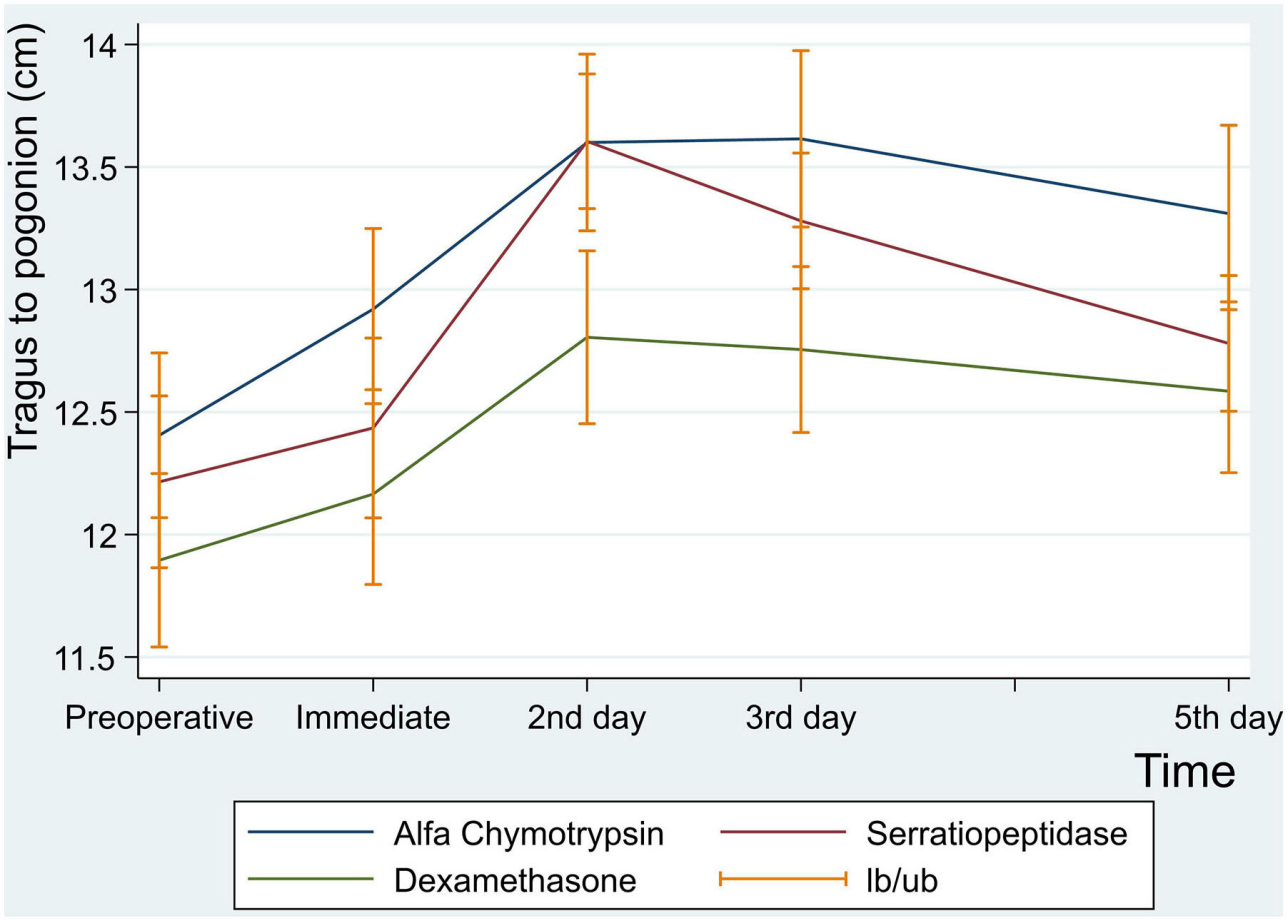
Facial swelling measurement for tragus to pogonion in centimeter at preoperative, immediate, 2nd , 3rd and 5th postoperative days.

There was no statistically significant difference between the three groups in postoperative mean value for the measurement of Tragus to Pogonion (*p* < 0.001) (Table 6 and Figure 4).

**Table 6:** Comparison of facial swelling (tragus to pognion) experienced by study groups.

	**Coefficient (95%) Conf. Interval**	***P*-value**
Group	−0.30 (−0.75 – 0.16)	0.199
A Vs B	−0.08 (−0.93 – 0.76)	0.85
A Vs C	−0.33 (−0.79 — 0.12)	0.15
B Vs C	−0.46 (−1.29 – 0.38)	0.28
Time	0.20 (0.10 – 0.30)	0.001
Group and time interaction	−0.02 (−0.07 – 0.02)	0.351

*A: Submucosal chymotrypsin group, B: Oral serratiopeptidase group, C: Dexamethasone group*.

#### Facial Swelling (Tragus to the Corner of the Mouth)

**Figure 5:**
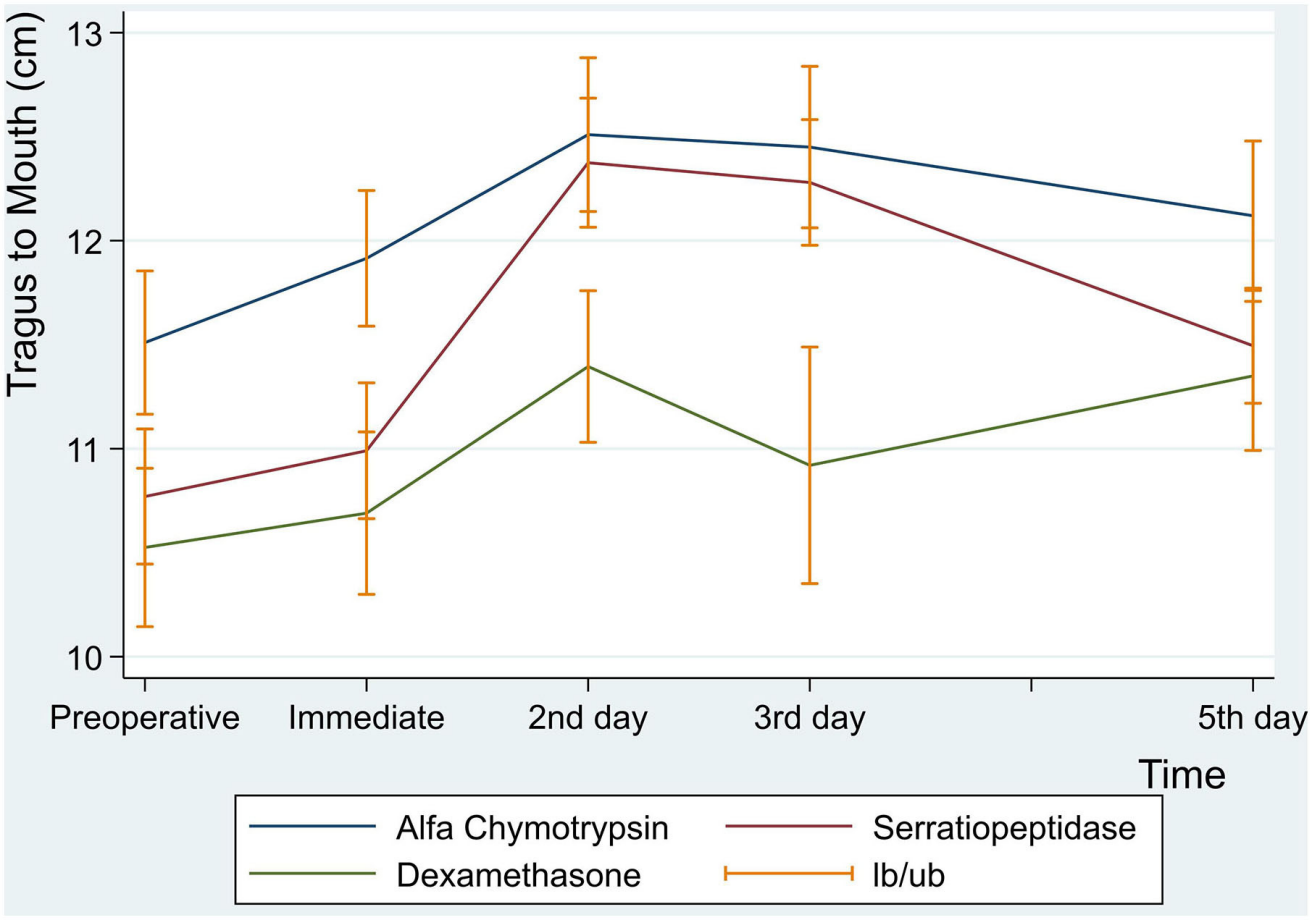
Facial swelling measurement for tragus to mouth in centimeter at preoperative, immediate, 2nd , 3rd and 5th postoperative days.

There were no statistically significant differences between the three groups in facial swelling measurement for Tragus to the Corner of the Mouth (*p* = 0.08) (Table 7 and Figure 5).

**Table 7:** Comparison of facial swelling (tragus to the corner of the mouth) experienced by study groups.

	**Coefficient (95% Conf. Interval)**	***P*-value**
Group	−0.59 (−1.07 – −0.11)	0.016
A vs. B	−0.38 (−1.26 – 0.49)	0.39
A vs. C	−0.63 (−1.14 – −0.12)	0.015
B vs. C	−0.75 (−1.70 – 0.21)	0.12
Time	0.076 (−0.085 – 0.236)	0.08
Group and time interaction	0.007 (−0.068 – 0.081)	0.856

*A: Submucosal chymotrypsin group, B: Oral serratiopeptidase group, C: Dexamethasone group*.

#### Facial Swelling (Gonion to Canthus)

**Figure 6:**
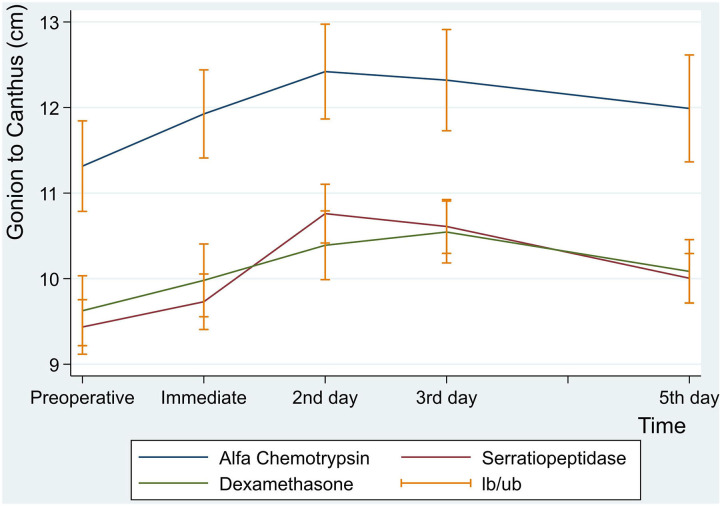
Facial swelling measurement for gonion to canthus in centimeter at preoperative, immediate, 2nd, 3rd and 5th postoperative days.

Similarly, there were no statistically significant differences between the three groups in facial measurement (Gonion to Canthus) (*p* < 0.001) (Table 8 and Figure 6).

**Table 8:** Facial swelling measurements (gonion to canthus).

	**Coefficient (95% Conf. Interval)**	***P*-value**
Group	−0.85 (−1.43 – −0.27)	0.004
A vs. B	−1.19 (−3.09 – −0.74)	0.001
A vs. C	−0.92 (−1.58 – −0.26)	0.006
B vs. C	−0.12 (−1.07 – 0.83)	0.8
Time	0.10568 (0.064 – 0.147)	< 0.0001
Group and time interaction	−0.032 (−0.051 – −0.013)	0.001

*Swelling (Gonion to canthus)*.

## Discussion

The present randomized clinical trial was aimed to assess an effectiveness of pre-operative sub-mucosal injection of 5 mg chymotrypsin and immediate postoperative 10 mg oral serratiopeptidase or 8 mg of oral dexamethasone following surgical extraction of the lower third molars in respect of postoperative sequalae (maximal mouth opening, facial swelling and pain intensity). The main key findings of the present RCT showed that there was a slight advantage at the first postoperative hour, 2nd, and the fifth postoperative days in the outcomes of pain intensity, facial swelling and maximal mouth opening for submucosal chymotrypsin over oral dexamethasone and serratiopeptidase. However, this advantage did not reach a significant level. Our findings were similar to study of Al-Sandook et al. who concluded that chymotrypsin administration for the third molar removal was associated with a significant reduction in the mean pain intensity scores at the 1st, 2nd, 3rd, and 7th postoperative days [30]. However, Al-Khateeb and Nusair compared between serratiopeptidase and placebo following lower third molars extraction, they found that Serratiopeptidase after surgical extraction of the third molar was associated with a significant reduction in the mean pain intensity scores at the 1st (*p* = 0.00), 2nd (*p* = 0.00), and 3rd (*p* = 0.00) postoperative days. So, their results were in disagreement with the presents results [9].

Similarities of achievement in reducing the postoperative sequalae after surgical extraction of impacted lower third molars among the three comparative groups (submucosal chymotrypsin and oral serratiopeptidase and dexamethasone) can be attributed to the relatively smaller sample size in each group (*n* =20), the absence of a placebo control group, inaccurate postoperative assessment methods (tape method for facial edema and VAS for pain intensity), patients incompliance and variation of patient's gender and age. Nevertheless, the three medications, namely submucosal chymotrypsin and oral serratiopeptidase or dexamethasone can have the same effectiveness in postoperative sequalae following surgical extraction of impacted lower third molars (as revealed by the present study).

Additionally, Kannan and Kavitha have investigated effectiveness of Serratiopeptidase in combination with Bromelain and diclofenac vs. diclofenac Sodium and conventional antibiotics or Bromelain alone after lower third molars surgery. They found that co-administration of Bromelain/Serratiopeptidase and diclofenac was significantly superior to diclofenac alone for the relief of pain up to 48 h (*p* < 0.05) [31]. However, because they combined Serratiopeptidase with Bromelain and diclofenac, the real effect of Serratiopeptidase cannot be identified effectively, and their results were inclusive.

Regarding maximal mouth opening during the different postoperative times, it has been observed in the current study that there was a sharp decrease in the maximal mouth opening starting from the first day, reached the lowest value at the second postoperative day, and then increased gradually up to the fifth postoperative day. These results are in consistent to Al-Sandook et al. However, the decrease in maximal mouth opening noticed in this study could be the results of the residual pain and inflammation, which were persisted during the 1st and 2nd postoperative days [30].

A systematic review and meta-analysis included 5 studies showing that a superior achievement in increasing maximal mouth opening following serratiopeptidase group when compared to corticosteroid, but a comparable insignificant difference with respect to facial swelling was found [32]. However, because this systematic review include 5 studies which, having wide heterogeneity and several confounding factors, they conducted meta-analysis for only 2 studies, thus, superiority of serratiopeptidase over corticosteroid following lower third molars surgery still need more studies before a final conclusion can be drawn. Anti-inflammatory effectiveness of serratiopeptidase can be explained by the enhanced viscosity of accumulated fluid predisposing drainage. Additionally, it has been proved that it can change cell-surface adhesion molecules that attract inflammatory cells to their target site [33]. Concerning analgesic efficacy, the pain reducing effect may be due to the inhibition of pain inducing bradykinin and other amines [34].

Meanwhile, a single randomized clinical trial showed significant pain reduction following serratiopeptidase compared to placebo following lower third molars surgery [9], patients used 1,000 mg paracetamol in combination with serratiopeptidase, thus, it cannot be certain if the pain reducing effects came from serratiopeptidase or paracetamol. Contrary to the preset study, patients received only predetermined specific medications based on type of group without any other medications (analgesic or antibiotic) that may act as confounding factors.

The main limitation of this study was: [1] the absence of a placebo group to compare a real effect of the three drugs. Thus, the results of the current study should be interpreted with caution.

Strengths of the current study were: [1] Randomization and allocation concealment were performed for all patients. Thus, election bias was prevented. [2] Blinding of both patients and assessor was conducted to eliminate performance and attrition bias. [3] Sample size calculation was performed to determine the power and significance level. [4] To the best of the authors' knowledge, this is the first randomized double blind clinical trial that assessed effectiveness and safety of submucosal injection of chymotrypsin following lower third molar surgery. [5] All surgical extractions were performed by one single surgeon, as well as outcomes assessment done by a blind assessor.

With the limitation of the present randomized double-blind, non-placebo, one can concluded that preoperative submucosal injection of chymotrypsin (5 mg) was a safe and effective drug in reducing postoperative pain intensity, facial swelling and maximal mouth opening following lower third molar surgery when compared to oral dexamethasone (8 mg) or oral serratiopeptidase. Further future randomized double-blind placebo control trials with larger sample sizes using magnetic resonance imaging or stereophotogrammetry to study facial swelling, are needing to assess the effectiveness of chymotrypsin, oral serratiopeptidase, corticosteroid vs. placebo (in different route and doses) in respect of postoperative sequalae following impacted lower third molars surgery.

## Data Availability Statement

The original contributions presented in the study are included in the article/supplementary material, further inquiries can be directed to the corresponding author/s.

## Ethics Statement

The ethical approval was obtained from the USTY Medical Research Ethics Committee (No. EAC/UST167) (Annex 1). Consent forms were taken from all patients who were free to accept or refuse their participation in the study.

## Author Contributions

EA-M: conception and design of study/review/case series: acquisition of data: laboratory or clinical/literature search, analysis and interpretation of data collected. Drafting of article and/or critical revision: final approval and guarantor of manuscript. AA-S: acquisition of data: laboratory or clinical/literature search; drafting of article and/or critical revision and final approval and guarantor of manuscript. EA-Z: acquisition of data: laboratory or clinical/literature search; drafting of article and/or critical revision: final approval and guarantor of manuscript. All authors contributed to the article and approved the submitted version.

## Conflict of Interest

The authors declare that the research was conducted in the absence of any commercial or financial relationships that could be construed as a potential conflict of interest.
